# Assessing the effectiveness of multi-session online emotion recognition training in autistic adults

**DOI:** 10.1371/journal.pone.0327424

**Published:** 2025-07-02

**Authors:** Zoe E. Reed, Oliver Bastiani, Andy Eastwood, Ian S. Penton-Voak, Christopher Jarrold, Marcus R. Munafò, Angela S. Attwood

**Affiliations:** 1 School of Psychological Science, University of Bristol, Bristol, United Kingdom; 2 MRC Integrative Epidemiology Unit, University of Bristol, Bristol, United Kingdom; 3 Psychology, School of Social Sciences, UWE Bristol, United Kingdom; 4 National Institute for Health Research Bristol Biomedical Research Centre, University Hospitals Bristol NHS Foundation Trust and University of Bristol, Bristol, United Kingdom; Northumbria University, UNITED KINGDOM OF GREAT BRITAIN AND NORTHERN IRELAND

## Abstract

Difficulties with emotion recognition can occur in neurodevelopmental conditions, including in autistic individuals. Providing interventions to support this would therefore be beneficial, particularly in terms of downstream effects on wellbeing, social relationships and education. In this online experimental study, we examined the effect of a recently developed facial emotion recognition training task versus a sham/control task in an adult population who self-identified as autistic over four sessions in a 2-week period, with a fifth follow-up session (N = 184). Our main analyses showed that facial emotion recognition accuracy was greater in Session 4 in the active group, with an estimated improvement of 14% (equivalent to approximately 7 additional correct responses), compared to 2% (equivalent to approximately 1 additional correct responses) in the sham group (*p* = 4x10^-09^). Additional analyses suggested training effects were generalisable to facial stimuli that participants had not been trained on and were still present, although attenuated, two weeks later. We also observed some self-reported improvements in social interactions post-training. Overall, this study demonstrated improved emotion recognition with this training task in an adult sample who self-identified as autistic. Future work is needed to investigate the effect of this task on emotion recognition accuracy in those with a formal diagnosis of autism, and in autistic children where support could be most beneficial.

## Introduction

Difficulties with emotion recognition (ER) – the ability to recognise other’s emotional expressions – occur across a range of neurodevelopmental conditions, particularly in autistic individuals or those with autistic traits – those scoring higher on autistic trait questionnaires [1–5]. The ability to recognise another’s emotional expressions is an important part of social cognition (i.e., processing social information and responding to this), which involves several processes (perception, processing of this information, and recognition of an emotion) [6]. For autistic individuals, difficulties could occur at any of these stages, but the recognition stage is a key point at which difficulties could be supported. Studies have demonstrated that there are *global* (rather than emotion-specific) difficulties across all emotions in autistic individuals [1]. In addition, response times for recognising emotions, in general, seem to be slower in autistic individuals compared to neurotypical individuals [5,7]. ER difficulties can negatively influence wellbeing and social skills, which in turn may negatively impact social relationships and educational outcomes such as school attendance [8–11]. Therefore, providing individuals with an intervention to help with ER is important and may have positive downstream benefits.

It is important to note that, historically, much of the research in this area has suggested that these differences are due to ‘deficits’ in autistic individuals. However, more recently there has been a shift in this viewpoint highlighting that these differences may, instead, be due to mutual misunderstandings between autistic and neurotypical individuals, known as the double-empathy problem [12,13]. For example, neurotypical individuals may struggle to understand how autistic individuals express and process emotional expressions and vice versa. Therefore, whilst research in this area suggests there are ER difficulties in autistic individuals, when considering how this influences real-world social interactions it is important to acknowledge the differences that autistic and neurotypical individuals may have in perceiving, processing and recognising emotions. It is also important to understand how emotions are processed by both autistic and non-autistic individuals to support difficulties that may be present in both groups.

There have been a number of training toolkits/interventions that have been developed targeting ER [14–18]. However, of these, many have either been developed to be delivered within a laboratory setting or with professional supervision which may limit accessibility compared to those available online or that do not require professional supervision [15,16]. Existing toolkits often do not have different levels of difficulty, i.e., different intensities of facial expression from more exaggerated to more subtle expressions of an emotion [14–16], or they have a limited evidence base [17] (including those that do not have any published research supporting them). In addition, many of the these existing toolkits/interventions focus on encouraging ‘correct’ interactions, reducing autistic traits, or are based on the notion that autistic individuals are less able to empathise than neurotypical peers [17,18], despite the recent shift away from ‘deficit’ focused viewpoints.

We have previously demonstrated effectiveness of a recently developed computer-based ER training task on emotion recognition accuracy in the general adult population and shown that the effects of this training transfer to facial stimuli other than those individuals were trained on [19]. This task presents facial emotional expressions, of varying intensities, and asks the individual to select the emotion they believe was presented. They are then given feedback as to whether this was correct or not and if incorrect, they try again until a correct response is given. This task is part of a wider toolkit being developed which will be delivered in the community, tailorable and designed to consider ER beyond the context of this task. This wider toolkit is different to exisiting interventions/toolkits in several ways. First, the stimuli used in this specific task have been created with different intensities of emotions, with more subtle expressions providing a greater level of difficulty. Second, this specific task is accessible online and requires no professional supervision. Third, the wider toolkit has been developed through co-design and involvement of autistic individuals and other stakeholders to ensure the toolkit is acceptable to the autistic community, and encourages mutual understanding of differences in emotion expression and emotion recognition. To ensure we provide an evidence-based task suitable for autistic individuals, it is important to determine whether similar training effects are observed in autistic individuals who may experience greater difficulties in this area and who would be the key user-group for interventions.

This online experimental study therefore examined the effect of an ER training task versus a sham/control task on ER in an adult autistic population (who self-identified as autistic). Previous work examined training during a single session; however, it is unclear whether training over multiple sessions may be of additional benefit. Therefore, this study comprised four sessions of training over a 2-week period. We hypothesised that participants randomised to ER training would show greater improvement in ER ability, after the 4 sessions, compared to those randomised to sham training. We also explored: 1) whether ER training effects transferred to other (untrained) facial stimuli; 2) whether ER training effects were observed for specific emotions; 3) whether there was evidence of continued ER improvement and impact on self-reported social interaction/skills two weeks after training completion; and 4) how participants in each group (ER training vs control task) subjectively found the training in terms of how engaging it was and views on usefulness to autistic individuals.

## Materials and methods

The protocol for this study was pre-registered on the Open Science Framework (https://osf.io/jszw7). Participants were recruited via the online recruitment platform Prolific (https://www.prolific.co/) and data collected via Gorilla, the online experiment builder (http://www.gorilla.sc/) [20].

### Ethics

This study received ethics approval from the School of Psychological Science Research Ethics Committee at the University of Bristol (approval code: 260821118826). Informed consent was obtained from participants through the study in Gorilla after being presented with the information sheet. Participants were provided with contact details of the researcher should they wish to contact them with any questions or concerns before continuing. Participants were asked to indicate in the online study whether they consented to take part or not. They were presented with the statement “I hereby fully and freely consent to my participation in this study as detailed above” after viewing the information sheet and consent form details. Participants could select “I consent” or “I do not consent”.

### Participants

A total of 220 participants were recruited and randomised to one of two training groups (active or sham) in a 1:1 ratio. Recruitment and study completion took place between 28^th^ September 2021 and 1^st^ April 2022. To be eligible, participants needed to be autistic (self-reported diagnosis of autism) or self-identified as autistic (self-reported on Prolific), be aged 18 years or over, and be fluent in English. In addition, they could not: be currently taking medication to treat a mental health condition or medication usually prescribed for this (as such medications have been shown to potentially influence performance on ER tasks [21,22]); have an uncorrected visual impairment, including colour vision deficiency; have participated in any related studies (https://osf.io/x4kh3, https://osf.io/drby2 and https://osf.io/bpzcj); or participated in fewer than 10 studies on Prolific (to identify Prolific users more likely to complete all 5 sessions). All screening questions were self-reported by participants in their Prolific profiles, with further confirmation from them in Gorilla to verify eligibility. The exact screening questions used in Prolific are provided in Section 1 in S1 File. There were no restrictions based on geographical location.

Sample size was guided by a previous study investigating effectiveness of an emotional bias retraining task that uses the same stimulus set as the ER task tested here [23]. An effect size of *d* = 1.08 in the balance point (i.e., bias score in a single emotion training study) was reported in that work. A more conservative effect size of *d* = 0.50 was used in our sample size calculation to account for the current task training six emotions simultaneously (compared to two in the bias version of the task) [24], and an extra 5% was added to the sample size due to potential attrition. At an alpha level of 5% (i.e., p = 0.05) for a two-tailed independent means t-test one would need 210 participants to provide 95% power to detect an effect size of *d* = 0.50. Therefore, we recruited a total of 220 participants via Prolific (accounting for the extra 5% needed).

However, not all participants met eligibility criteria when asked the screening questions in Gorilla (N = 20) and therefore these individuals were excluded from analyses (post-randomisation). In addition, we excluded participants who did not have complete data for Session 4 (N = 9) or had outliers in their data for total hits at baseline or in Session 4 (N = 7). Therefore, 184 individuals were included in the analyses (94 in the active and 90 in the sham groups).

### Study procedure

**Fig 1:**
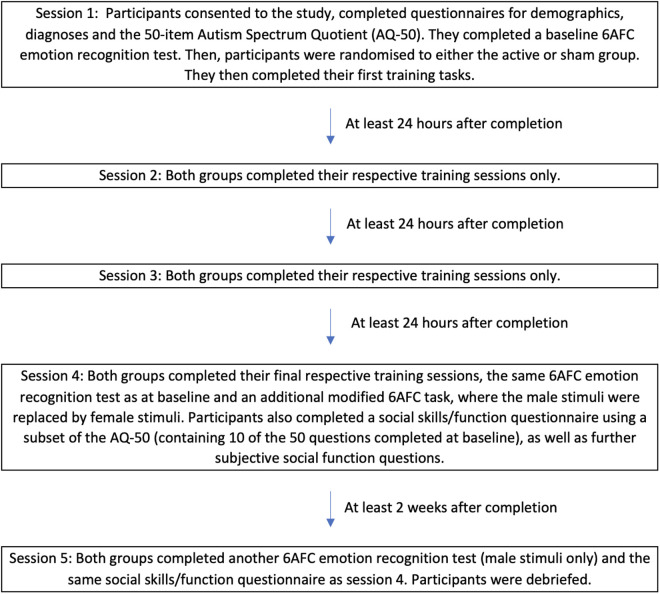
Study session overview. 6AFC=Six alternative forced choice (emotions were angry, happy, sad, scared, surprised and disgusted).

The study consisted of a total of 5 sessions to assess the effect of ER training over time, with 4 of these being training sessions and the primary outcome (total number of correct responses on the ER task) assessed after training in session 4. The 5 sessions were completed over a 3–4-week period, with each of the first 4 sessions completed at least 24 hours after the previous session and Session 5 completed approximately 2 weeks after Session 4 (see Fig 1). If any participants did not complete session 2 by the end of day 12, they were not invited back for session 3 and they were replaced (N = 4). In addition, if a participant did not complete all 4 sessions they were replaced (N = 6). These individuals are not included in the final total sample size of 184.

### Demographic information

Demographic information on age, gender and education were collected in Session 1. For gender, participants were asked ‘What gender do you identify as?’ and they could select from male, female, and non-binary. For education, participants were asked ‘What is the highest level of education you have completed?’ and they could select from ‘Degree or equivalent higher education and above’, ‘A level or equivalent’ (A levels: these are UK subject specific qualifications that are typically completed over 2 years between the ages of 16 and 18), ‘GCSEs grades A*-C or equivalent’ (General Certificate of Secondary Education: UK subject-specific qualifications typically completed over 3 years towards the end of secondary school education), ‘No qualification’, or ‘I don’t know’. The last two options were combined for analysis.

### Emotion recognition test – six alternate forced choice (6AFC)

The ER test was included in Sessions 1 (baseline), 4 (primary outcome) and 5 (follow up – secondary outcome). On each trial, participants were presented with a single facial image (the same white male face for all). These stimuli were computer-generated by averaging photos of 12 individuals, and therefore do not show an identifiable person [25]. Each stimulus expressed one of six emotions (happy, angry, sad, scared, surprised, and disgusted) at one of 8 levels of intensity (neutral to 100% of that emotional expression). Therefore, there were a total of 48 trials, which were each displayed once and were shown in a random order. Facial emotion expression images were presented on screen for 150 milliseconds (ms), before being masked for a further 250ms, and then participants proceeded to the next screen with the six emotions displayed as words. Here, participants were asked to select the word that they thought represented the displayed emotion, with the selection screen remaining present until participants had made their choice. After the choice was made no feedback was provided and participants moved onto the next image, preceded by a fixation cross. Further details of this test can be found in our previous publication [19].

### Generalisability test

In Session 4, post-training, participants completed a further ER test (with the same parameters as the main ER test), but with white female facial stimuli instead. The purpose of this was to test whether effects of training would generalise to non-trained faces.

### Emotion recognition training task

**Fig 2:**
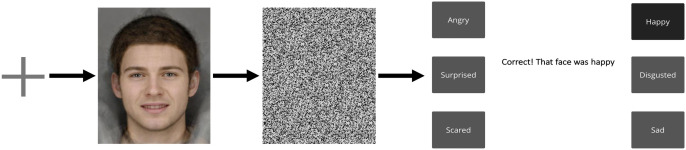
Example of ER test task.

The active training group completed a modified version of the ER test, whereby the procedure remained the same, with exceptions that each face was displayed for 1000ms, and participants selected an emotion word until they were correct. Feedback was presented to participants after each selection, and they could only proceed to the next face image once they had answered correctly. Fig 2 shows an example of the ER test task and further details can be found in our previous publication [19].

### Sham training task

For the sham training group, a similar training task was completed, however instead of faces with emotional expressions, the stimulus images presented were of coloured rectangles (blue, red, green, yellow, purple and orange, all ranging from grey to the full colour with 8 increments), with participants being asked to select which colour they had seen displayed on the preceding screen. They were also provided with feedback, and they could only proceed to the next image once they had answered correctly.

### Emotion recognition outcome measures

The primary outcome for statistical analyses was the total number of correct responses, i.e., the number of times participants correctly selected the emotion corresponding with the facial expression displayed (hits). This was used as an indicator of ER accuracy in the baseline and post-training (Session 4) tests.

Outcomes for secondary analyses were 1) total hits from the post-training generalisability test, 2) total hits in Session 5, approximately 2 weeks post-training (i.e., to examine effects after a time delay), 3) emotion-specific sensitivity scores (using the signal detection parameter A-prime (A’) index which is a non-parametric estimate of discriminability) [26] post-training in Session 4 (analyses were also conducted for hits and false alarms – the total number of times an emotion was selected when this was not the correct response – which are factored into the sensitivity scores and which are presented in S1 File Hits and false alarm outcomes across all analyses were converted to proportions.

### Autism and mental health diagnoses

As not all participants recruited had a self-reported diagnosis of autism (some self-identified as autistic or were going through the diagnosis process), participants were asked whether they had ever received a formal diagnosis of autism to allow us to explore whether this had an impact on the results. Participants were also asked whether they had a diagnosis of anxiety, depression, or other mental health issue to assess whether co-occurring mental health conditions impacted results.

### Autistic traits

The full 50-item Autism Spectrum Quotient (AQ-50) questionnaire was included to measure autistic traits [27]. The total possible score was 50, with higher scores indicating more autistic traits. For each statement in the AQ-50, participants were asked to choose the response that best describes them from the following options: ‘Definitely Agree’, ‘Slightly Agree’, ‘Slightly Disagree’ or ‘Definitely Disagree’. The full AQ-50 is provided in Section 2 in S1 File.

A 10-item subset of the Autism Spectrum Quotient (see Section 2 in S1 File) was used to measure social skills specifically and data from this were collected in Sessions 4 and 5 (as well as part of the full AQ-50 in Session 1). The score on this subset of questions was used to assess whether there were any changes in social skills following training.

### Other social skills questions

In Sessions 4 and 5, additional questions were asked to measure any subjective changes in social skills not picked up by the social skills subset of the AQ-50. Participants were asked ‘Has the frequency of your social interactions increased since participating in this study?’ and ‘Do you feel that this study has improved your ability to recognise other people’s emotions?’. These were rated on a scale of 0–100 where 0 indicated “not at all” and 100 indicated “very much so”.

### Subjective ratings of training

To assess whether there were group differences in how they perceived the study participants were asked whether they found the tasks tiring and interesting and whether the instructions were easy to follow. They were also asked whether they thought the ER training would be useful for autistic individuals. The exact questions asked are provided in Section 3 in S1 File. These were rated on a scale of 0–100 where 0 indicated “not at all” and 100 indicated “very much so”.

### Statistical analysis

#### Primary analysis.

All analyses were conducted in R version 4.0.2 [28]. Prior to analysis we removed individuals whose total hits scores were outliers in the baseline and Session 4 post-training tests (i.e., data points that fell 1.5 times above or below the interquartile range). Then data were also assessed for normality using skewness and kurtosis statistics. To assess the hypothesis that participants randomised to ER training would show greater improvement in ER ability after the 4 sessions we used a linear mixed effects (LME) model to compare group differences (active versus sham training) for total hits (the number of correct responses), accounting for between participants random variance, with variables of time (baseline and Session 4 – the primary outcome), group, and an interaction term for time x group. This was conducted using the lme4 package in R [29]. Random intercepts for each participant ID were included for the random effects. We ran the following models: 1) an unadjusted model, 2) a model adjusted for age, gender and education level (as fixed effects), and 3) a model additionally adjusted for scores on the AQ-50 at baseline.

#### Secondary analyses.

We conducted several secondary analyses to examine generalisability to non-trained stimuli, maintenance of effects over time, emotion-specific effects and wider impacts of ER training (i.e., on social skills and subjective ratings of the training).

First, to explore whether ER training effects transferred to other (untrained) facial stimuli we ran a similar model to our primary analysis but the outcome for Session 4 was hits on the generalisability test.

Second, we examined whether there was any evidence that training effects were maintained after approximately 2 weeks by running a similar LME model to that for the primary analysis but instead using hits data from Session 5 instead of Session 4.

Third, to examine whether ER training effects were observed for specific emotions we ran analyses to explore sensitivity scores across the individual emotions (as well as hits and false alarm rates). This was achieved by running LME models for each emotion for sensitivity scores (or hits or false alarms) as outcomes with the same variables as in the adjusted primary outcome model.

Fourth, to assess whether there was any transfer of ER training effects onto social skills after Session 4 and two weeks after training completion (Session 5) we also ran similar LME models to those in the primary analysis (adjusted), but instead using the AQ-50 social skills subset as the outcome for Session 4 and for Session 5, as two separate models.

Fifth, exploration of the data from the other subjective social skills questions asked in Sessions 4 and 5 was conducted using t-tests to compare the groups to examine how participants in these groups subjectively found the training. We also assessed whether each of the subjective ratings of training experiences (i.e., sham vs active) varied between the two groups by conducting two-tailed independent means t-tests and calculating Cohen’s d to estimate the effect size of the group differences.

Other pre-registered exploratory analyses are provided in Section 4 in S1 File.

#### Sensitivity analyses.

We conducted additional sensitivity analyses for our primary analyses and secondary analyses of hits in Session 5 by 1) excluding participants with any other mental health diagnosis to see whether having a co-occurring mental health diagnosis impacted total hits, given that previous studies have demonstrated emotion recognition difficulties and biases for some mental health conditions [30,31], 2) excluding participants who had encountered technical issues during the study (e.g., completing a session over multiple days or 2 on the same day, which could have impacted their outcome) and 3) including participants with outliers in their data (N = 7) for total hits at baseline or in Session 4 (the primary outcome measure) to assess whether results were different when including these individuals. Individuals were considered to have data with outliers where their total hits scores at baseline or Session 4 post-training tests fell 1.5 times above or below the interquartile range).

## Results

### Participant characteristics

Table 1 shows a description of the sample. Of note, the mean AQ-50 score at baseline was similar across the two groups with a mean of 34.24 (SD = 6.97) in the active group and 33.32 (SD = 6.97) in the sham group. These mean values are above the previously described ‘clinical’ threshold of 32 [27]; however, only 64% of the active group and 57% of the sham group are above this threshold, suggesting that despite the participants self-identifying as autistic there are some that may not meet clinical thresholds. However, the number of participants who reported a diagnosis of autism did vary between the groups with fewer reporting a diagnosis in the sham (29%) compared to the active group (48%). In addition, whilst the percentage of males was similar between the groups there were fewer females in the sham group due to a higher number of non-binary participants compared to the active group. Other baseline characteristics and performance on the baseline ER task were similar across the two groups. After removing outliers, we examined skewness and kurtosis for baseline and post-training (Session 4) total hits. Histograms of these distributions are shown in S1 Fig in S1 File. Skewness and kurtosis measures were within an acceptable range (see Section 5 in S1 File for further details).

**Table 1:** Participant characteristics.

		Active group (N = 94)	Sham group (N = 90)
Mean age in years (SD)		28 (9)	29 (11)
Gender (%)	Male	48%	48%
	Female	51%	44%
	Non-binary	1%	8%
Education (%)	Degree or equivalent	56%	49%
	A-levels of equivalent^1^	29%	34%
	GCSEs (grades A* to C) or equivalent^2^	7%	9%
	None or unsure	7%	8%
Diagnosis of autism (%)		48%	29%
Co-occurring mental health diagnosis (%)		57%	66%
Mean total hits (SD)	Baseline	0.63 (0.09)	0.62 (0.10)
	Session 4	0.76 (0.09)	0.64 (0.10)
	Generalisability test in session 4	0.73 (0.08)	0.67 (0.09)
	Session 5	0.74 (0.10)	0.66 (0.11)
Mean AQ-50 score at baseline (SD)		34.24 (6.98)	33.32 (6.97)
Mean social skills AQ-50 subscale score (SD)	Baseline	6.96 (1.81)	6.00 (1.96)
	Session 4	6.83 (1.88)	6.43 (2.03)
	Session 5	6.93 (1.83)	6.38 (2.09)

SD = standard deviation, AQ-50 = 50-item Autism Spectrum Quotient. ^1^A-levels or Advanced level qualifications are subject specific qualifications in the UK that are typically completed over 2 years between the ages of 16 and 18 (although can be completed over different time periods and different ages), ^2^GCSEs (General Certificate of Secondary Education) are subject-specific qualifications in the UK that are typically completed over 3 years towards the end of secondary school education; A* was the highest possible grade.

### Primary analysis results: emotion recognition accuracy in session 4

**Fig 3:**
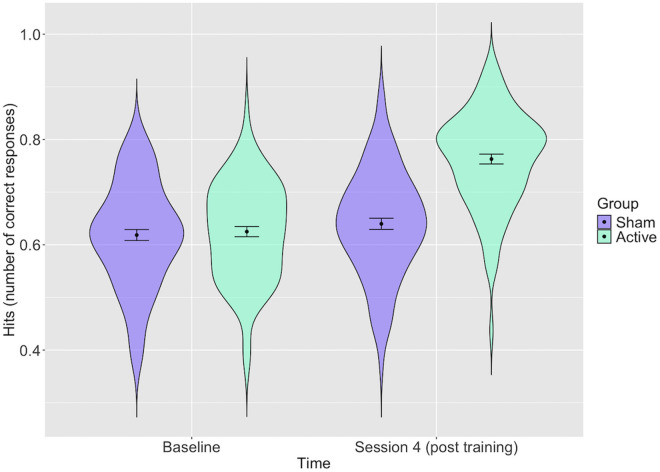
Distribution of participants’ total hits with estimates for the active and sham groups at baseline and post training (Session 4). *Error bars represent 95% confidence intervals: Distribution of participant’s scores (proportion of total correct hits) with estimates for each group before and after training and confidence intervals shown. The active group shows greater improvement post-training*.

Analyses indicated that the active group showed greater improvement post-training in Session 4 compared to the sham group. Specifically, the interaction model revealed that the proportion of total hits was greater in Session 4 in the active group compared to the sham group (see Fig 3 and S1Table in S1 File) in the unadjusted model and the model including age, gender and education level (including covariates: *b* = 0.12, 95% CI = 0.08 to 0.16, *p* = 4x10^-09^). In the fully adjusted model, the sham group hits increased from an estimated 67% at baseline to 69% in Session 4, whilst the active group increased from an estimated 67% to 81%. Results were similar in the model that additionally included scores on the AQ-50 at baseline (see S1 Table in S1 File).

### Secondary analysis results

#### Generalisability.

**Fig 4:**
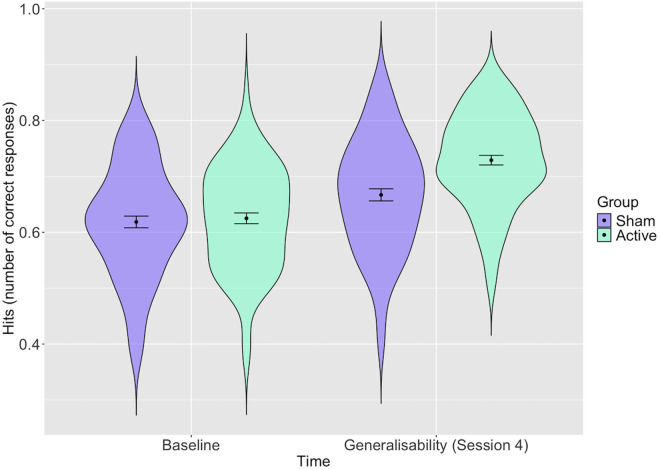
Distribution of participants’ total hits with estimates for the active and sham groups at baseline and in the post training generalisability test. *Error bars represent 95% confidence intervals: Distribution of participants scores (proportion of total correct hits) with estimates for each group before and after training and confidence intervals shown. The active group shows some improvement post-training*.

In the generalisability test (Fig 4 and S3 Table in S1 File) we found slightly attenuated results but there was still a clear indication of greater hits post-training in the active group than the sham group (*b* = 0.06, 95% CI = 0.02 to 0.09, *p* = 0.005), with the sham and active group’s hit count increasing from an estimated 67% to 72% and 78%, respectively.

#### Maintenance.

**Fig 5:**
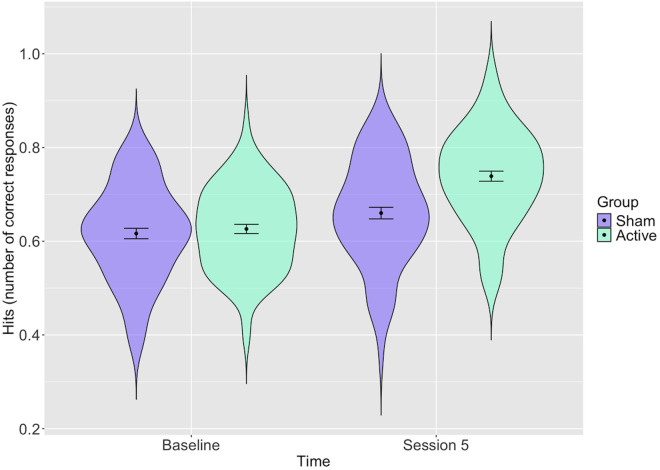
Distribution of participants’ total hits with estimates for the active and sham groups at baseline and 2 weeks post training (Session 5). *Error bars represent 95% confidence intervals: Distribution of participants scores (proportion of total correct hits) with estimates for each group before and after training and confidence intervals shown. The active group shows greater improvement post-training*.

There was some attenuation of the training effect at Session 5 (2 weeks post-training) for the active group (Fig 5 and S4 Table in S1 File), but this still remained, indicating that this selective improvement persisted over time (*b* = 0.07, 95% CI = 0.03 to 0.11, *p* = 0.001), with the sham and active group’s hit count increasing from an estimated 67% and 68% to 71% and 79%, respectively.

#### Emotion specific models.

Results from the LME models examining emotion-specific sensitivity scores, hits and false alarm rates are presented in S5 to S7 Tables in S1 File. For sensitivity scores the was evidence of interaction effects between time and group for all emotions except surprised and disgust, where the active group showed higher scores (indicating greater discriminability) compared to the sham group post-training. For hits there was evidence of interaction effects between time and group for all emotions except disgust. This indicated that the active group recognised all emotions except disgust better than the sham group post-training. Finally, for false alarms there was evidence of interaction effects between time and group for the emotions of scared, surprised and disgusted, where the active group had fewer false alarms for these emotions compared to the sham group post-training.

#### Social skills.

**Fig 6:**
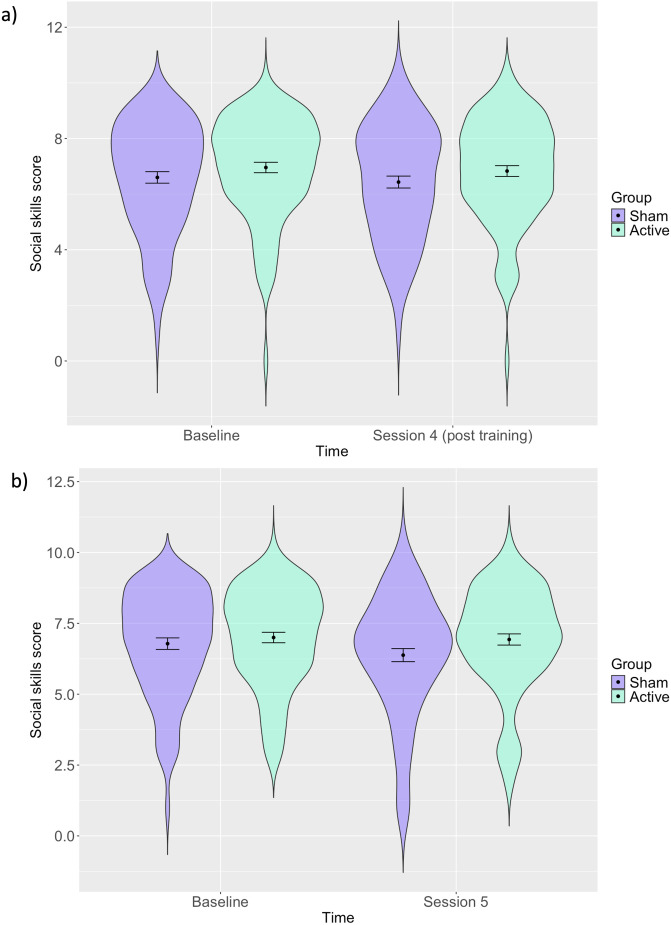
Distribution of social skills as measured by a subset of the AQ-50 at baseline and post-training in Session 4 (a) and Session 5 **(b).** *Error bars represent 95% confidence intervals: Distribution of participants social skills scores with estimates for each group before and after training and confidence intervals shown. There are no differences pre and post training for both groups*.

The results from LME models examining whether there was any transference of ER training effects onto social skills (as measured using a subset of the AQ-50) indicated that there was no meaningful difference in the active group compared to the sham group post-training after Session 4 (*b* = 0.04, 95% CI = −0.31 to 0.39, *p* = 0.83) (Fig 6a), or Session 5 (*b* = 0.32, 95% CI = −0.01 to 0.66, *p* = 0.06) (Fig 6b and S8 Table in S1 File).

#### Subjective ratings.

We found that there were some group differences in subjective ratings of the study which were measured out of 100 (S9 Table in S1 File). In particular, the active group found the study slightly more interesting (active mean = 62 [SD = 27], sham mean = 52 [SD = 25], Cohen’s d = −0.38 [95% CI: −0.67 to 0.08], *p* = 0.01) and easier to follow (active mean = 96 [SD = 9], sham mean = 90 [SD = 15], Cohen’s d = −0.48 [95% CI: −0.78 to −0.19], *p* = 0.002) than the sham group, but there were no meaningful differences observed for how tiring the study was (active mean = 48 [SD = 27], sham mean = 45 [SD = 29], Cohen’s d = −0.10 [95% CI: −0.39 to 0.19], *p* = 0.51). Results from both groups suggest that ER training may be useful for autistic individuals and this measure was rated similarly across the two groups (active mean = 69 [SD = 23], sham mean = 68 [SD = 21], Cohen’s d = −0.02 [95% CI: −0.31 to 0.27], *p* = 0.88). In addition, differences were found between the groups for the question relating to ability to recognise other people’s emotions at the end of Session 4 (i.e., higher in the active group) (active mean = 40 [SD = 27], sham mean = 26 [SD = 23], Cohen’s d = −0.56 [95% CI: −0.86 to −0.27], *p* = 0.0002) and this was rated even higher after Session 5 (active mean = 47 [SD = 24], sham mean = 33 [SD = 22], Cohen’s d = −0.62 [95% CI: −0.93 to −0.31], *p* = 9.19x10^-05^), although we note that values were low in the overall measure out of 100. Additionally, although meaningful group differences in self-reported frequency of social interactions after Session 4 were not observed (active mean = 20 [SD = 26], sham mean = 17 [SD = 33], Cohen’s d = −0.12 [95% CI: −0.41 to 0.17], *p* = 0.40), a meaningfully greater frequency of social interactions was seen after Session 5 in the active compared to the sham group (active mean = 38 [SD = 30], sham mean = 28 [SD = 28], Cohen’s d = −0.33 [95% CI: −0.64 to −0.02], *p* = 0.03).

### Sensitivity analysis results

Results were similar for the sensitivity analyses, in terms of magnitude, direction and strength of evidence of an effect, compared to the primary analyses with Session 4 total hits (on the ER task) as the outcome and secondary analyses with Session 5 total hits (on the ER task) as the outcome. This was the case when conducting the analyses1: 1) excluding participants with any other mental health diagnosis and 2) excluding participants with who had encountered technical issues during the study and 3) including participants with outliers in their data (N = 7) for total hits at baseline or in Session 4 (S2 and S4 Tables in S1 File).

## Discussion

We examined whether active ER training improved ER of facial expressions compared with sham training in adults who self-identified as autistic. Our results indicate an estimated improvement of 14% (equivalent to approximately 7 additional correct responses) in the active group compared to 2% (equivalent to approximately 1 additional correct responses) in the sham group in our adjusted models, demonstrating effectiveness of ER training in self-identified autistic individuals. These results build on our previous study in a non-autistic sample [19]. In the present study we observed a greater overall improvement in ER compared to a previous study which included only one session. This may indicate that a greater number of sessions is more beneficial, but, as the samples were different, further studies are needed to confirm this.

A limitation of this study was the use of a single facial stimuli in the main training and test tasks. Therefore, we also examined the generalisability of this ER training. Although our results were slightly attenuated in the generalisability test, we still observed an effect suggesting transference of training to novel facial stimuli. It should be noted that the generalisability test was conducted with only one additional set of facial stimuli (a white female face) and therefore further testing of generalisability with a wider range of stimuli (including different ages and ethnicities) should be included in future work. Similarly, our results were attenuated, but with an effect still observed, approximately 2 weeks after the last training session indicating that there is a lasting effect of the training. It is unclear how long this effect may be sustained for and therefore further studies with longer follow-up periods would be useful.

The emotion-specific analyses revealed some group differences after training, particularly in the cases of disgust, scared, and surprised where the training had less of an effect than for the other emotions. In general, most previous research in this area suggests that autistic individuals experience global ER difficulties as opposed to emotion specific difficulties [1,3,7,32]. There is some evidence to suggest that specific emotions, in particular disgust, may be more difficult to recognise for autistic individuals [33]. In addition, other evidence suggests that recognition of happiness in autistic individuals does not differ from neurotypical individuals [4]. However, multiple meta-analyses have concluded that there is a lack of evidence supporting differences for specific emotions and therefore our findings for specific emotion differences should be interpreted with caution [1,3,7]. If there are emotion specific differences, it could be that they are heterogenous amongst autistic individuals, potentially reflecting biases due to other co-occuring difficulties where emotion specific difficulties have been found. For example, ER biases are present in mental health conditions such as depression [31,34], and the possibility of such biases could be explored further in autistic individuals with co-occurring conditions. It may therefore be the case that further and more tailored training is needed where emotion specific difficulties are present.

We did not observe any meaningful differences in the social skills measure post-training, although this is not necessarily surprising given that the social skills measure is a subset of the autistic traits measure. In addition, it is important to note that whilst the AQ-50 is a widely used measure within research studies, it is limited in several ways [35–37]. First, using a subset of the AQ-50 is likely to mean that potentially important questions relating to autistic traits are not included, therefore potentially resulting in important aspects of autistic traits being missed. Second, previous research shows that context and reference groups (i.e., who the participant is considering when answering such questionnaires) matter and scoring is likely to differ based on these factors. Third, such measures of autistic traits are unlikely to be as valid in non-autistic individuals; this is an important consideration given that there may have been individuals in the study who would not meet diagnostic thresholds. Nevertheless, we did observe some evidence of group differences post training on greater self-reported ER improvements and social interactions in the active group, which were even more apparent 2 weeks after the training.

Overall, our results are in line with previous studies demonstrating improved ER after training [38–40]. Our study also suggested effects are maintained two weeks post-training, whereas a previous meta-analysis suggested that effects were not maintained, although follow-up times were variable and not included in all studies, so their conclusions were limited [39]. Most of the previous studies in this area did not find evidence to suggest social skills improved post training, in line with our findings [39,40]. However, our self-reported responses suggest there might be some improvement from a subjective perspective, suggesting that this should be examined in greater depth in future studies. There is limited information on generalisability in previous studies [39,40]. Therefore, it is difficult to compare our generalisability results to previous studies, confirming that this is an area that requires further study. Our results suggest that this task would be useful to include in future research in this area.

There are a number of existing interventions targeting ER in autistic individuals as outlined in the introduction [14–18]. The task presented here adds to those in the previous literature in several ways. First, through this study and previous studies [19] we have established a good evidence base for this task in terms or improvements in ER and generalisability. This task is also deliverable in an online setting which means that it can easily be utilised across a range of contexts and does not require professional supervision to complete. Finally, this task includes different levels of difficulty and therefore has the potential to be incorporated into a wider tailorable and co-created toolkit.

### Limitations

Our study, whilst conducted in a well-powered sample over multiple sessions, is subject to some limitations. First, the stimuli used in all sessions were of the same individual; a decision taken to avoid making the task too long, particularly given its use over multiple sessions. As a result, although we found that effects were generalisable to other non-trained facial stimuli this needs to be tested for other facial stimuli (e.g., facial stimuli with different ages and ethnicities). Second, we are unable to determine whether the effects observed are due to mere exposure effects (of faces) – individuals becoming more familiar with the facial stimuli as opposed to the training component of the task influencing emotional processing ability. Exposure effects are not necessarily problematic if the result is still ER being supported in the real world. In addition, previous studies using the same facial stimuli for bias retraining (which similarly has a feedback component) demonstrate that training effects transfer to untrained facial stimuli [25,41]. Third, this work similarly cannot distinguish the mechanisms behind any improvements in ER and it is important to consider that autistic individuals may have a different approach to emotion recognition than neurotypical individuals and that there will likely be differences within the autistic population, i.e., not all autistic individuals will experience ER difficulties.

Fourth, a limitation of our study is that we conducted the study online (due to the COVID-19 pandemic). This may impact our findings in several ways: i) we recruited from individuals signed up to Prolific which likely resulted in a selected sample, therefore future work would ideally be conducted by engaging individuals from across the community, ii) we cannot be sure how well these results generalise to real world settings (e.g., with children in a classroom), so this would need to be examined further and iii) there is no way to really verify who has taken part in the study beyond Prolific’s checks or how well the participants engaged/paid attention to the study. This latter point is particularly crucial given the recent increase in fraudulent participants in online studies [42]. However, by using a platform such as Prolific for recruitment, where participants must verify their accounts by providing photo ID and accounts are checked regularly for signs of suspicious activity, we hope that the potential for fraudulent participants to have taken part in our study was minimised. Although, future studies with in-person data collection would also be useful to confirm this study’s findings, by conducting the study online we were able to allow autistic individuals to take part in a more accessible way. Fifth, our results may be limited by the fact that a large proportion of the sample did not have a formal diagnosis of autism. We included those who self-identified as autistic to be more inclusive in our research, however this may mean that those who would not meet diagnostic criteria are included. Therefore, we are unable to make very strong conclusions about the relevance of this task to those with a confirmed autism diagnosis. Furthermore, the percentage of participants who reported a diagnosis of autism was lower in the sham group than the active group. However, the mean AQ-50 score was similar across groups and performance on the task at baseline did not seem to differ. Finally, although we screened for colour vision deficiencies it may be that a participant is unaware that they have a colour vision deficiency, and therefore this may have impacted the sham task with colours. Given that this was a sham task this is unlikely to influence our results for ER, but alternative tasks could be considered in the future avoiding the use of colours.

### Future directions

This study demonstrated improvement in ER post training. Further studies would be useful to examine the extent of the training in more detail. For example, comparable studies with additional stimuli including different genders, ages and ethnicities would be useful to further explore generalisability. Future work should also examine differences in ER within autistic populations in order to create tasks that are tailorable to individuals as opposed to using a ‘one size fits all approach’, particularly through co-design with autistic individuals. Similarly, given the recent criticisms of social skills training which highlights potential downstream negative consequences of such training (e.g., not feeling able to be authentic [43], it is important that any toolkits/interventions using these types of tasks are developed with input from autistic individuals to ensure they are acceptable and useful to the autistic community. Also, In this study we worked with self-identified autistic adults (i.e., some individuals did not have a diagnosis). Therefore, future work with individuals with a confirmed autism diagnosis will be important to establish whether findings translate to this group. In addition, future work with children would be useful, as childhood is likely where most individuals would need support in this area, and where positive impacts on downstream outcomes would be more likely. Furthermore, we excluded individuals taking medications for mental health conditions as they may have influenced performance on the ER tasks. However, future studies examining the impact of medications on this task would be useful. It should also be noted that we had 10 participants start the study who then did not complete all 4 sessions and were therefore replaced. This is a small proportion of the overall number who did take part, but it would be important to ensure that the task is engaging in future work. Finally, further studies with other validated social interaction measures, which the autistic community consider to be useful to examine, and which consider the context and reference groups when using these measures, would be beneficial in order to ascertain whether there are improvements in these areas. Future research is needed to i) determine the optimal number of sessions, because in the current study, the number of hits continued to increase over sessions, and it is unclear how this would change over further sessions, ii) examine the downstream impact of ER training beyond just improving ER. Future research in this area should be conducted with input from the autistic community to create ER tasks which truly support autistic individuals who choose to receive support in this area.

## Conclusions

Overall, we found that multi-session ER training improved ER in an adult sample who self-identified as autistic. We additionally observed ER improvements that remained over time, and transferred to novel facial stimuli, and which may have a positive impact on social engagement and self-reported ER. Although further work is needed to determine: 1) whether one would see these improvements in adults with a confirmed diagnosis of autism and autistic children, and 2) if there is transference to further stimuli and the real-world emotions, and whether improvements in ER have further downstream impacts, this study provides a good evidence base for this form of training task. It therefore provides a basis for further development of ER training tasks to support autistic individuals with ER difficulties.

## Supporting information

S1 File (DOCX)
